# Protocol for fabricating immune-regulatory bone organoids via plant-based bioinks

**DOI:** 10.1016/j.isci.2026.117866

**Published:** 2026-10-12

**Authors:** Tianhao Wang, Gan Gao, Xiaoxu Zhang, Kaifeng Huo, Chenglong Liu, Zhen Geng, Long Bai, Hao Zhang, Han Liu, Ke Xu, Yuxiao Lai, Jianhua Wang, Yingying Jing, Chongru He, Guangchao Wang, Jiacan Su, Xiaoxiang Ren

**Affiliations:** 1 Organoid Research Center, Institute of Translational Medicine, Shanghai University, Shanghai 200444, China; 2 MedEng-X Institutes, National Center for Translational Medicine (Shanghai) SHU Branch, Shanghai University, Shanghai 200444, China; 3 School of Medicine, Shanghai University, Shanghai 200444, China; 4 Department of Orthopedics, Xinhua Hospital, Shanghai Jiao Tong University School of Medicine, Shanghai 200092, China; 5 Suzhou Innovation Center of Shanghai University, Suzhou 215000, China; 6 The Second Hospital of Sanming City, Sanming 353000, China; 7 Centre for Translational Medicine Research & Development, Institute of Biomedical and Health Engineering, Shenzhen Institute of Advanced Technology, Chinese Academy of Sciences, Shenzhen 518055, China

**Keywords:** bone organoids, Aloe vera, immune modulation, macrophage polarization, 3D bioprinting

## Abstract

Bone organoids offer three-dimensional systems for studying skeletal development, disease, and regeneration. However, many existing bone organoid systems do not adequately incorporate immune-regulatory cues, despite their central role in bone repair. Their translational potential is further limited by the use of animal-derived matrices such as Matrigel, which are xenogeneic, poorly defined, and subject to batch-to-batch variation. To address these limitations, we developed a Matrigel-free and exogenous cytokine-free bioink composed of Aloe vera gel (AV), gelatin methacryloyl (GelMA), and Laponite. This formulation supports the fabrication of immune-regulatory bone organoids by providing both a printable extracellular matrix-like scaffold and intrinsic immune-regulatory signals. *In vitro*, the bioink promotes sustained macrophage reprogramming and helps establish an immune microenvironment that supports bone organoid maturation. Here, we describe a step-by-step protocol for preparing the plant-containing bioink, fabricating immune-regulatory bone organoids, and evaluating their performance in cranial bone defect models. This defined and reproducible platform provides a practical alternative to conventional Matrigel-based systems and may be adapted for the engineering of other immune-responsive tissues.

## Highlights

• Matrigel-free aloe bioink confers intrinsic immune-regulatory cues
• Natural aloe components support prolonged M2 macrophage polarization
• The composite hydrogel optimizes 3D shape fidelity and bone matrix assembly
• Engineered bone organoids bridge immune regulation and skeletal defect repair

## Graphical abstract

**Figure:**
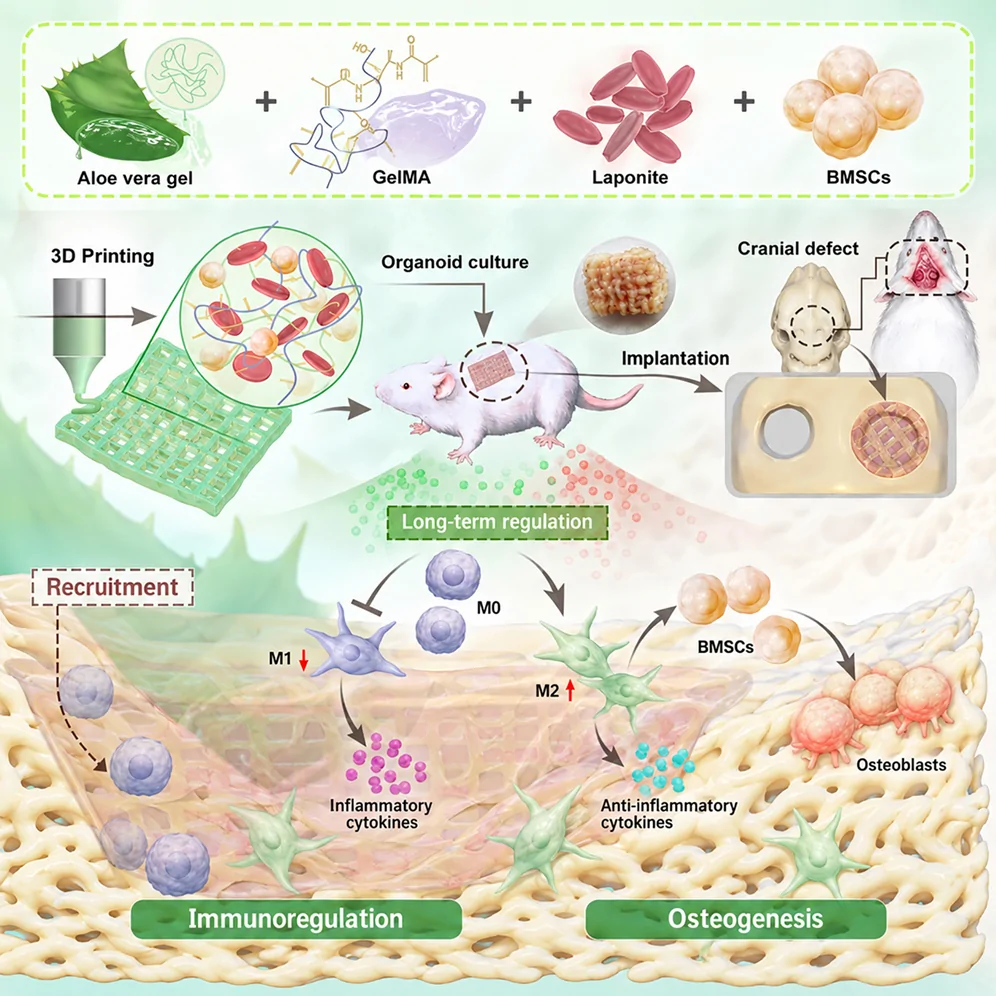

## Teaser

Biological sciences; Applied sciences

## Introduction

Over the past decades, organoids have attracted considerable interest in developmental biology, disease modeling, and drug screening, largely because these three-dimensional structures, derived from pluripotent or adult stem cells, can closely mimic key structural and functional features of native tissues. Unlike classical epithelial organoids that primarily rely on spontaneous self-organization, engineered bone organoids may require biomaterial support to reproduce the mineralized and mechanically dependent bone microenvironment. Here, we define scaffold-guided engineered bone organoids as constructs in which biomaterials establish the initial architecture, while encapsulated cells subsequently drive matrix remodeling, bone-specific extracellular matrix deposition, and progressive mineralization. These dynamic biological processes distinguish them from passive cell-laden scaffolds.^1^ Bone organoids provide a promising strategy for studying bone development and promoting the repair of large defects.^2^^,^^3^^,^^4^ Nevertheless, current bone organoid platforms are largely designed to support osteogenic differentiation and matrix production, with limited integration of immune components. This represents a significant drawback, given that immune cells, especially macrophages, are known to play a crucial role in functional bone regeneration. During the healing process, macrophages typically shift from a pro-inflammatory (M1) state to a pro-regenerative (M2) state. This phenotypic transition helps resolve inflammation, induce new vessel formation, promote osteogenic differentiation, and facilitate extracellular matrix remodeling. Developing immune-regulatory bone organoids may therefore improve the physiological relevance and regenerative potential of these systems.

Current organoid culture protocols commonly rely on Matrigel as the basal scaffold. Derived from murine sarcoma cells, this basement membrane mimic supplies the three-dimensional structural support required for organoid growth and maturation. However, its animal origin introduces several inherent drawbacks, including a complex and poorly characterized composition, undefined biochemical constituents, and significant lot-to-lot variability. These factors undermine the reliability and reproducibility of experimental results and create major obstacles for the clinical translation of organoid-based technologies.^5^^,^^6^ There is therefore a pressing need to develop alternative hydrogel matrices that are more defined, reproducible, and suitable for translational development (Table 1).

**Table 1:** Comparative analysis of the GelMA/Laponite/AV bioink system versus conventional Matrigel-based organoid platforms

Feature	Conventional Matrigel-based systems	GelMA/Laponite/AV bioink system
Composition	complex, incompletely defined tumor-derived ECM	Matrigel-free and compositionally tunable; GelMA and AV require batch-specific quality control
Reproducibility	susceptible to lot-to-lot variation	improved control through defined formulation ratios and standardized material characterization
Immunomodulation	not specifically designed for controlled immune regulation	AV-associated regulation of macrophage recruitment and polarization
Printability	Matrigel alone generally exhibits limited shape fidelity and structural stability	extrusion-printable, shear-responsive, and photocrosslinkable
Mechanical properties	relatively soft and difficult to tune independently	adjustable through GelMA concentration, methacrylation degree, Laponite content, and photocrosslinking conditions
Translational potential	limited by tumor origin, compositional variability, and regulatory concerns	avoids Matrigel and offers improved manufacturing control, but requires further standardization, biosafety evaluation, and scale-up validation

To address these challenges, we developed a Matrigel-free and exogenous cytokine-free bioink containing gelatin methacryloyl (GelMA), Laponite, and Aloe vera (AV) gel , with the goal of generating bone organoids that combine structural support with immune-regulatory activity.^7^ The bioink consists of three ingredients: GelMA, synthetic Laponite nanoclay, and AV. GelMA in combination with Laponite provides the structural scaffold that supports bone organoid formation and promotes osteogenic differentiation. In this system, the Laponite particles adopt a house-of-cards microstructure, which enhances the mechanical strength of the hydrogel and enables 3D printability. Meanwhile, AV functions as the immunologically active component, delivering a range of natural bioactive compounds that guide long-term macrophage polarization. This effect helps sustain immune regulation within the surrounding tissue environment, a feature critical for replicating native tissue dynamics.

In this work, we describe an approach that uses plant-containing bioinks to fabricate bone organoids with immunomodulatory capacities. We also provide a detailed, step-by-step protocol for applying these organoids in animal models of calvarial bone defects. This well-defined, Matrigel-free, and reproducible platform offers a scalable alternative to Matrigel and opens a promising path for the *in vitro* engineering of other immune-sensitive tissues, beyond the skeletal system (Figure 1).

**Figure 1:**
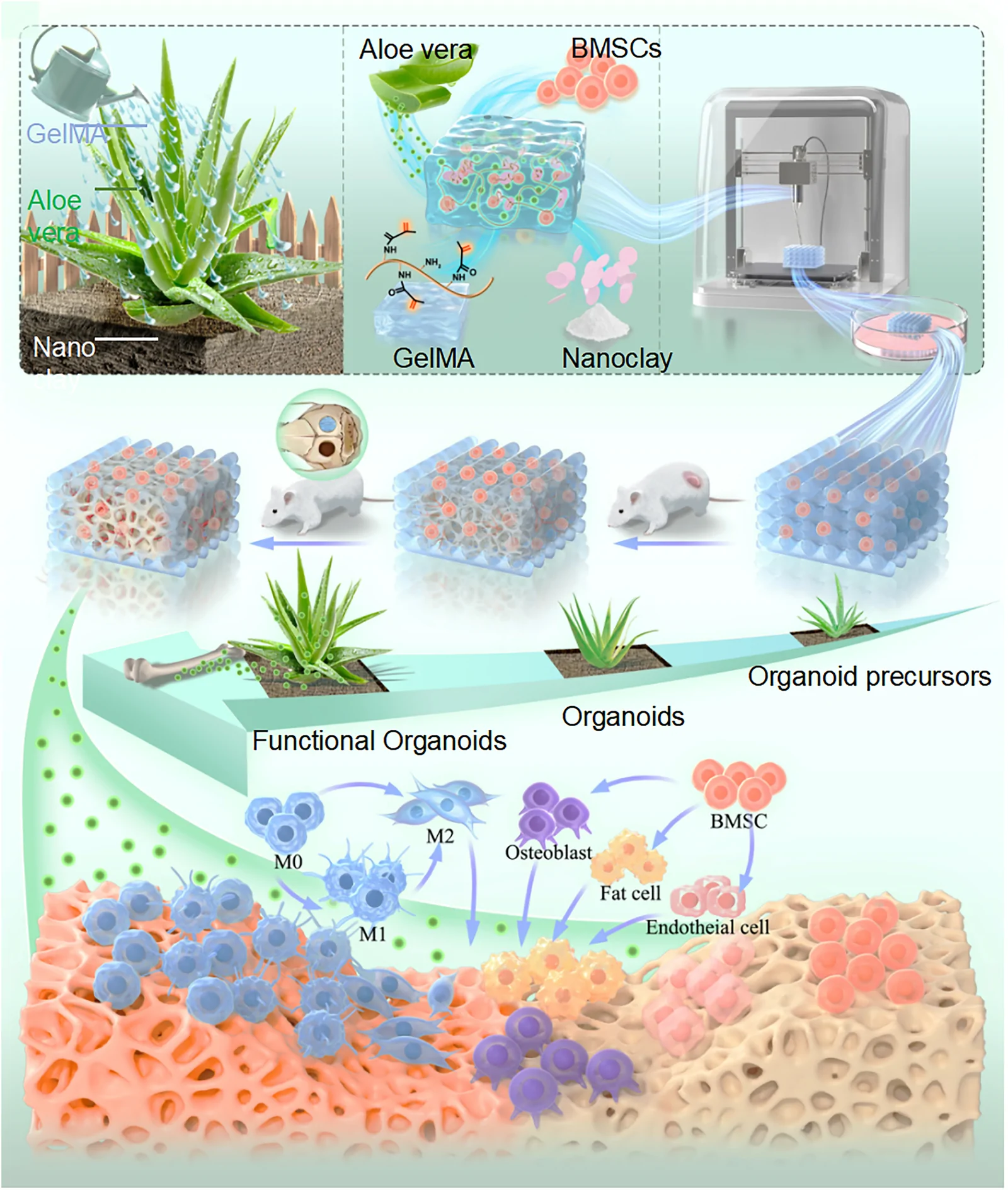
Schematic illustration of bone organoid composition, their role in facilitating defect recovery, and the dual mechanisms of immunomodulation and osteogenesis

## Results

### Experimental design and workflow

This protocol describes the fabrication of immune-regulatory bone organoids using a plant-based composite bioink (GelMA/Laponite/AV), *in vitro* characterization of the immunomodulatory guidance and osteogenic development and *in vivo* validation of the ability to repair critical-sized bone defects applying bone organoids.

In brief, the composite bioink was prepared by uniformly blending UV-sterilized GelMA, Laponite, and AV at the optimized ratio of 10%:1%:40% (GelMA:Laponite:AV). Concurrently, bone marrow mesenchymal stem cells (BMSCs) were encapsulated within this bioink at a density of 1 × 10^6^ cells/mL, yielding the cell-laden composite bioink. Subsequently, extrusion-based 3D bioprinting was performed using the following parameters: a printing temperature of 8°C, an extrusion pressure ranging from 0.2 to 0.3 MPa, 5% UV light intensity, a print speed of 4 mm/s, a nozzle retraction height of 2 mm, 1.5 mm infill distance, a 90° interlayer rotation repeated twice, and a 21G metal nozzle. The printed constructs were then cross-linked under UV light (365 nm) to form bone organoid precursors. After printing, the bone organoid precursors were immediately transferred to a static culture environment maintained at 37°C and 5% CO_2_. Initially, the samples were cultured for 24 h in minimum essential medium alpha (α-MEM) supplemented with 10% fetal bovine serum. Following this, the culture medium was switched to osteogenic induction medium supplemented with β-glycerophosphate, ascorbic acid, and dexamethasone; the cells were then maintained for up to 28 days, with the medium refreshed every 2–3 days. On day 14 and 28, the progress of the osteogenic differentiation was assessed. This was done with alkaline phosphatase (ALP) staining, Alizarin Red S (ARS) staining, mineralization analysis, quantitative reverse-transcription PCR (RT-qPCR) and western blot analysis. The capacity of the organoids to induce M2 macrophage polarization was also evaluated with macrophage migration, invasion, adhesion assays, and the shift toward the M2 phenotype.

To evaluate the functional maturation and immune regulation *in vivo*, the precursors of bone organoids were subcutaneously implanted into nude mice to examine ectopic osteo-immunological remodeling and vascularization (CD31^+^), analyzed by micro-computed tomography (CT), histology, immunofluorescence, and transcriptomic sequencing (RNA sequencing, RNA-seq). Finally, the bone organoids were evaluated in a rat model with a critical-sized calvarial defect to test their ability to promote functional bone repair, accompanied by biosafety evaluations including organ histology and blood biochemistry. GelMA-only and GelMA/Laponite groups served as control conditions throughout all experimental evaluations, alongside a blank control group for the calvarial defect model.

### Development and parameter optimization of plant-based bioinks

The protocol was developed by selecting each bioink component for a defined function. GelMA, a photocrosslinkable derivative of gelatin, was chosen as the primary scaffold material. Owing to the presence of the arginine-glycine-aspartic acid (RGD) motif, GelMA supports cell adhesion, proliferation, and matrix interactions, while offering favorable biodegradability and biocompatibility. Its mechanical properties can be conveniently tuned through rapid photo-cross-linking, making it suitable for providing structural support during stem cell differentiation.^8^^,^^9^ Laponite nanoclay was incorporated to improve the rheological behavior and structural integrity of the bioink. Its characteristic “house-of-cards” microstructure imparts strong shear-thinning properties, which are particularly advantageous for extrusion-based 3D bioprinting.^10^ Moreover, Laponite particles exhibit high surface area and excellent dispersibility, contributing to enhanced mechanical strength of the hydrogel composite. Notably, degradation products such as Mg^2+^ and Si^4+^ have been shown to create a pro-regenerative microenvironment that supports bone formation, further justifying the inclusion of Laponite in the scaffold formulation to improve printability and augment mechanical properties such as elastic modulus and toughness. AV was incorporated as the immune-regulatory component. Liquid chromatography-mass spectrometry (LC-MS) analysis identified multiple small-molecule constituents, including phytosphingosine- and polyphenol-related metabolites, while AV is also known to contain bioactive polysaccharides such as acemannan. These constituents may contribute to macrophage regulation and tissue repair.^11^ Unlike approaches that rely on exogenous cytokines such as IL-4, the AV-containing bioink produced sustained macrophage recruitment and pro-regenerative polarization in the original study.^12^^,^^13^ Bulk RNA-seq revealed broad immune remodeling involving Th1/Th2/Th17-related pathways and alterations associated with STAT6, NF-κB, PI3K/AKT, and RANKL-related signaling. These findings are consistent with enhanced pro-regenerative responses and reduced inflammatory signaling.

After selecting the appropriate constituents for the composite bioink, the optimization of their respective proportions was performed through a systematic multi-parametric evaluation balancing printability, structural fidelity, mechanical properties, and post-printing cell viability. Printability was quantified through filament extrusion continuity, diffusion ratio (Dfr), and the printability coefficient (Pr). Cell survival was assessed via Live/Dead staining and CCK-8 proliferation assays on encapsulated BMSCs, while structural robustness was determined by measuring compressive Young’s modulus. Each constituent’s concentration was optimized based on defined trade-offs: GelMA at 10 wt% provided the baseline cell-adhesive hydrogel network; lower concentrations resulted in weak mechanical support and structural collapse, while higher concentrations restricted cell migration and matrix remodeling. Laponite at 1 wt% acted as the key rheological driver; its “house-of-cards” microstructure imparted strong shear-thinning behavior, elevating shape fidelity (Pr ≈ 1.0), whereas higher Laponite loading caused nozzle clogging and elevated shear stress that reduced cell viability below 80%. AV at 40 vol% supplied the optimal threshold of bioactive polysaccharides (e.g., acemannan) for robust, long-term M2 macrophage polarization; lower AV fractions weakened immunomodulatory efficacy, whereas higher AV fractions over-diluted the precursor solution and compromised photo-cross-linking structural integrity. Consequently, the final optimized formulation of GelMA:Laponite:AV = 10%:1%:40% with a BMSCs density of 1 × 10^6^ cells/mL was selected as the sweet spot, yielding continuous filament deposition, precise 3D architecture, and superior cell viability exceeding 90%.

We further optimized the extrusion-based 3D bioprinting parameters to improve structural accuracy and minimize adverse effects on cell viability during the fabrication process. It was found that keeping the printing temperature low (8°C) is essential to keep the gel-like consistency of the composite bioink, as the reduced temperature helps prevent filament deformation and facilitates layer-by-layer construction.

The pneumatic pressure was set in a range of 0.2–0.3 MPa, and the printing velocity was maintained at 4 mm/s to ensure uniform and reliable filament deposition while avoiding excessive shear forces that could compromise cell viability. For UV cross-linking, a light intensity of 5% was applied to provide instant mechanical reinforcement to the printed structure and to reduce the risk of cellular damage from long time UV irradiation. In short, the optimized printing parameters were as follows: printing temperature of 8°C, pneumatic pressure of 0.2–0.3 MPa, 5% UV intensity, and the associated printing operation steps. A printing speed of 4 mm per second was used. The retraction height was 2 mm, the fill spacing was 1.5 mm, and the interlayer rotation angle was 90°, and it was done two times. A metal nozzle of the gauge 21 was used. With these specific settings, a rectangular scaffold was obtained. This structure was 5 mm in length and width, and built with eight different layers. It was designed as a precursor of a bone organoid, and this fabricated construct was put into a standard *in vitro* culture environment, at 37°C, with 5% carbon dioxide.

Subsequently, a series of *in vitro* assays were carried out to characterize the cultivated bone organoids. Osteogenic differentiation and matrix mineralization were evaluated using alkaline phosphatase and Alizarin Red S staining, mineralization quantification, RT-qPCR, and western blot analysis. The immune-regulatory effects were assessed through macrophage migration, infiltration, adhesion, iNOS/CD206 phenotyping, and analysis of IL-1β and IL-10 expression. For *in vivo* evaluation, bone organoid precursors were subcutaneously implanted into immunodeficient nude mice and analyzed using micro-CT, H&E staining, immunofluorescence, and RNA-seq. type 1 collagen, periostin, and vinculin were examined to assess extracellular matrix deposition and cell-matrix interactions, while CD31 staining was used to identify vascular-associated structures. The constructs were further evaluated in a rat calvarial-defect model for up to 8 weeks. The GelMA/Laponite/AV constructs promoted a CD206-positive macrophage response, supported BMSC osteogenic differentiation, and enhanced bone-matrix deposition and mineralization. Following implantation, they developed bone-like tissue and interacted with recruited macrophages and host vascular cells. These dynamic osteogenic-immune-vascular interactions and cell-driven matrix remodeling distinguish the scaffold-guided engineered bone organoids from passive cell-laden scaffolds. To our knowledge, this immunomodulation-driven strategy for achieving functional osteogenesis has not been reported previously (Figure 2).

**Figure 2:**
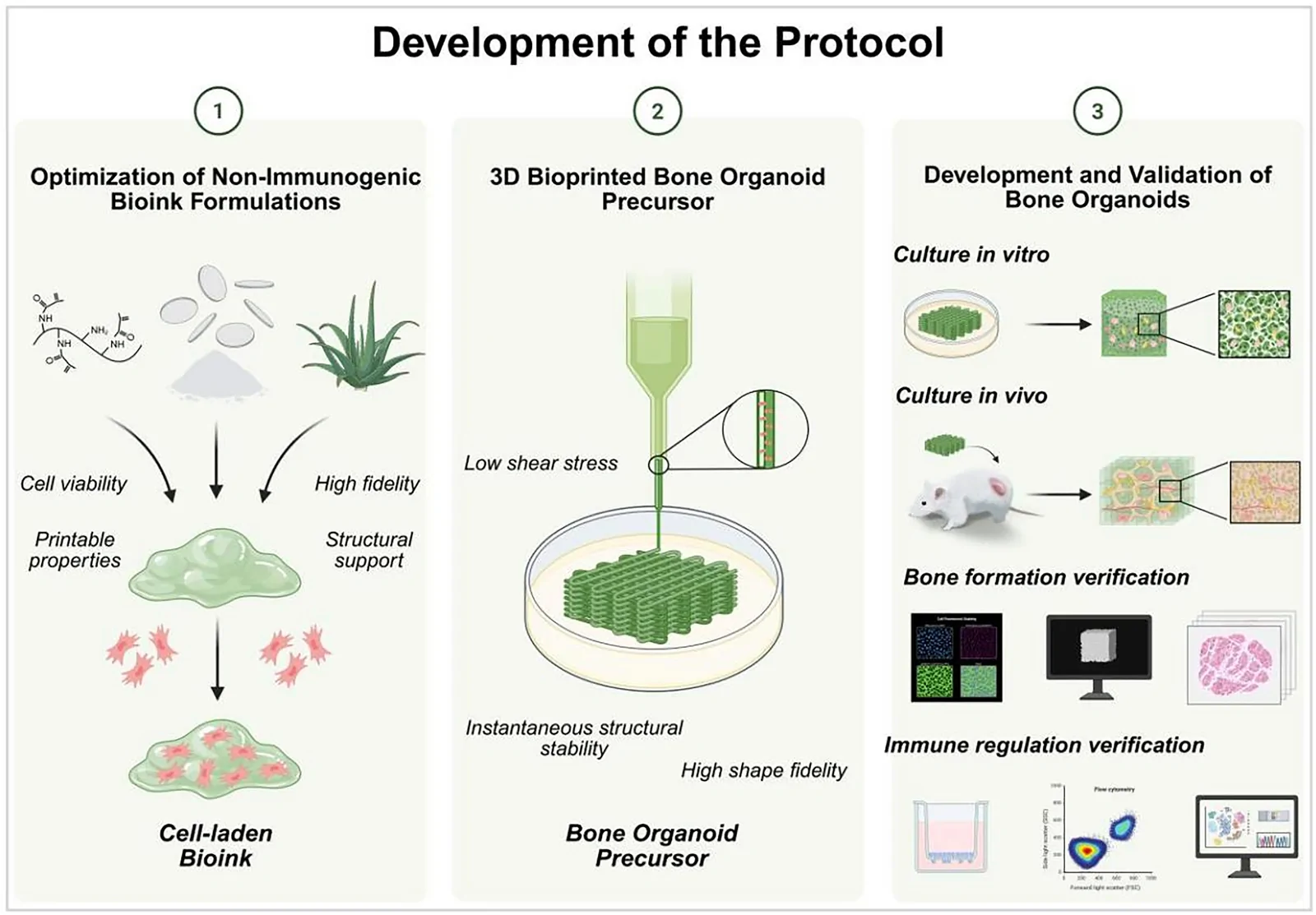
Development of the protocol for constructing immune-regulatory bone organoids

### *In vitro* and *in vivo* evaluation of immune-regulatory bone organoids

Using the bioink that contains GelMA, Laponite and AV (GelMA/Laponite/AV) (Figure 3B), bone organoid constructs are expected to be obtained within 14–28 days (Figure 3A). *In vitro*, successful constructs are expected to maintain high cell viability after printing (Figure S1A), exhibit high structural fidelity (Figure S1B), and to show progressive osteogenic differentiation and extracellular matrix deposit. These outcomes are reflected by ALP expression (Figure S2B), ARS-positive mineral deposition (Figure S2A), increased expression of osteogenic markers (e.g., OCN, RUNX2, and OPN), and micro-CT-detectable mineralized matrix formation. In extended cultures, researchers may further evaluate late-stage maturation using osteocyte markers (e.g., DMP1 and SOST) and lacuno-canalicular network imaging. With respect to immunomodulation, GelMA/Laponite/AV constructs are expected to increase macrophage recruitment and adhesion and to shift macrophages toward a CD206-positive, iNOS-low phenotype. These responses should be interpreted in parallel with appropriate GelMA and GelMA/Laponite controls.

**Figure 3:**
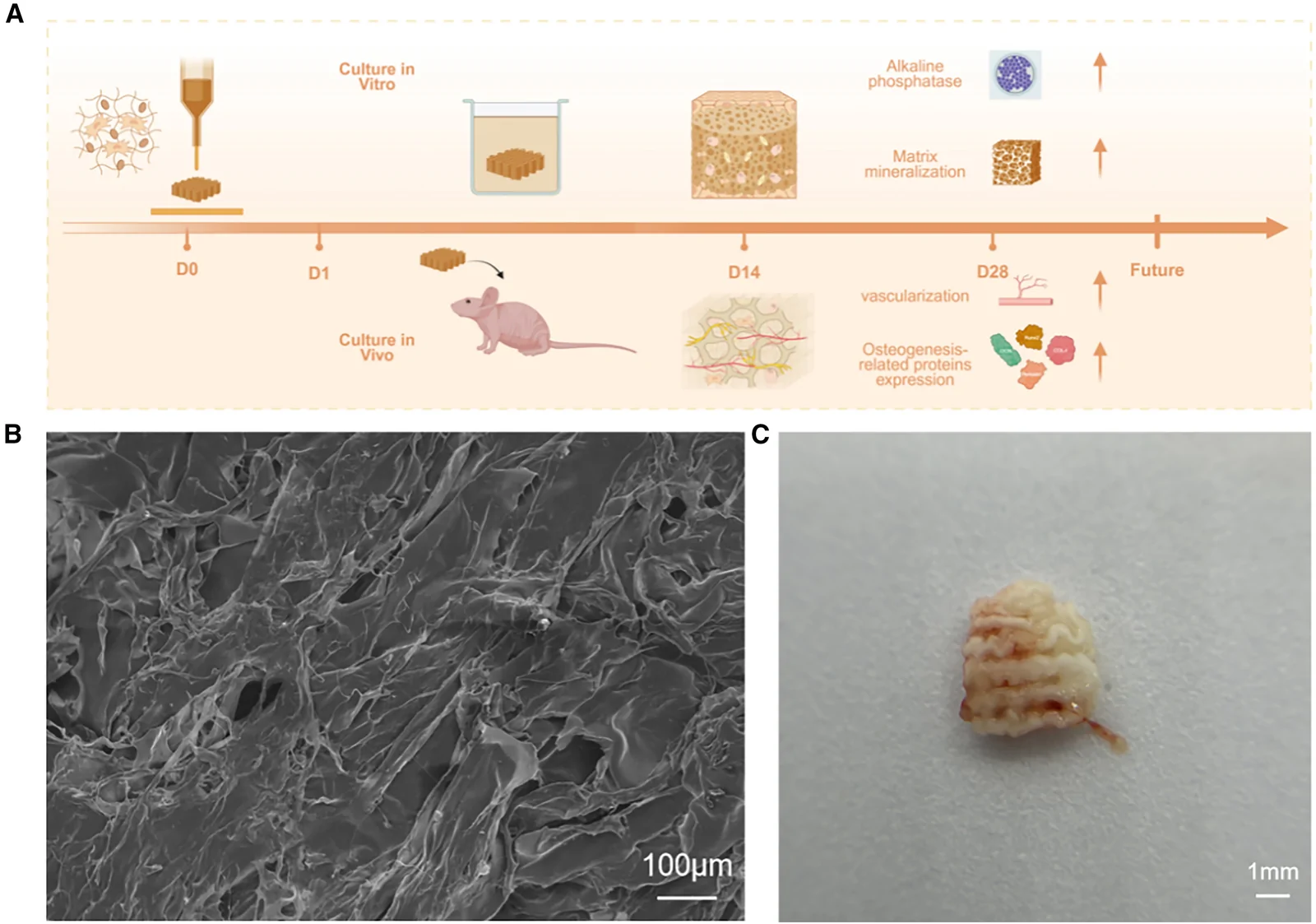
Construction of bone organoids via Matrigel-free plant-based bioink *in vitro* and *in vivo* (A) Schematic illustration of the bone organoid construction workflow. (B) Scanning electron microscopy image of Aloe vera gel (scale bar =100 μm). (C) Bright-field image of bone organoids after 28 days of *in vivo* development (scale bar = 1 mm).

After subcutaneous (s.c.) implantation, these constructs are expected to develop into bone-like tissues and undergo further maturation. Organoid maturation and electron cryomicroscopy (ECM) remodeling are reflected by organized extracellular matrix assembly (type 1 collagen and periostin), active cell-matrix focal adhesions (vinculin), high expression of osteogenic markers such as OCN, RUNX2, and OPN, and micro-CT-detectable mineralized matrix formation (Figures S2C and S2D). Additionally, bone organoids display macroscopic vascular-associated features, including prominent surface capillary infiltration and internal CD31-positive microvascular staining (Figure 3C). The immune-regulatory response is characterized by reduced IL-1β expression, increased IL-10 expression, and a shift toward a CD206-positive macrophage phenotype.

In the rat model featuring critical-sized bone defects, GelMA/Laponite/AV bone organoids should promote more robust bone regeneration compared with control scaffolds. By 8 weeks, the newly formed tissue is expected to show improved mineralized bone formation, closer integration with host tissue, and positive staining for osteogenic markers such as OCN and RUNX2. Importantly, CD206-positive macrophages within the repair site would further support the presence of a pro-regenerative immune microenvironment. Under the conditions described, H&E staining of major organs and routine blood biochemical analysis are anticipated to reveal no obvious systemic toxicity.

## Discussion

### Bioactive cues and signaling mechanisms in osteo-immunomodulation

A distinctive feature of this platform is the incorporation of AV to establish an immune-instructive microenvironment that supports bone organoid maturation. In the original study, these pro-regenerative effects were associated with modulation of Th1/Th2/Th17-related pathways and alterations in STAT6-, NF-κB-, PI3K/AKT-, and RANKL-related signaling. These results suggest potential pathways underlying AV-associated immune regulation but do not establish direct causality. Macrophage-specific transcriptomics, phosphorylation assays, pathway inhibitors, and characterization of individual AV components will be required for mechanistic confirmation. Furthermore, while the 8-week outcomes in rodent defect models effectively validate early osteo-immunological events, defect bridging, and acute biosafety, full clinical translation requires continuous, long-term matrix remodeling. Future applications of this protocol are therefore encouraged to extend evaluation timelines beyond 8 weeks (e.g., 12–24 weeks) in preclinical models. Such extended tracking will allow researchers to systematically monitor complete hydrogel degradation kinetics, dynamic bone mineral density progression, long-term microvascular network stabilization, and biomechanical load-bearing integration, thereby providing deeper insights into the long-term efficacy and safety of engineered bone organoids.

### Methodological considerations and troubleshooting

The reproducible fabrication and functional evaluation of immune-regulatory bone organoids require stringent quality control across several critical operational stages. During bioink formulation and 3D printing, maintaining a constant low temperature (8°C) and verifying the complete exfoliation and dispersion of Laponite nanoclay prior to adding AV are essential to prevent filament collapse, nozzle clogging, or non-uniform cell distribution. For cell-laden constructs, minimizing hydrodynamic shear forces during extrusion and avoiding excessive ultraviolet cross-linking are imperative to preserve post-printing stem cell viability above 90%. Furthermore, batch-to-batch consistency of AV is critical to ensure prolonged pro-regenerative macrophage polarization, while sufficient and verified EDTA decalcification of mineralized specimens is required to obtain intact histological sections. The potential procedural failure modes, their underlying causes, and practical corrective solutions are systematically summarized in Table 2.

**Table 2:** Troubleshooting of immune-regulatory bone organoid fabrication and analysis

Problem	Possible reason	Suggested solution
Discontinuous or collapsed printed strands	bioink temperature is too high, Laponite is incompletely dispersed, or extrusion pressure is not matched to viscosity	re-cool the cartridge and stage to 8 °C, gently remix the bioink, remove bubbles, and adjust pressure within the optimized range before printing a new construct
Reduced post-printing cell viability	excessive shear stress, prolonged handling, or unnecessary UV exposure	use gentle mixing, avoid repeated loading and unloading of the cartridge, maintain the optimized pressure and speed, and minimize UV exposure to the validated setting
Weak macrophage M2 polarization	AV gel activity varies between batches or the AV fraction is not evenly distributed in the bioink	record AV batch information, confirm gel homogeneity before printing, and include GelMA and GelMA/Laponite controls in each experiment
Low mineralization during osteogenic culture	cells are not healthy at printing, osteogenic medium is not refreshed regularly, or constructs are damaged during medium change	use early-passage BMSCs with high viability, replace osteogenic medium every 2–3 days, and handle constructs gently during medium changes
Poor histological section quality	insufficient decalcification or incomplete embedding of mineralized constructs	extend EDTA decalcification as needed, check tissue softness before sectioning, and orient constructs consistently during embedding

### Potential future applications and extensions of the protocol

The GelMA/Laponite/AV bioink was developed for fabricating immune-regulatory bone organoids. By combining a printable hydrogel network with a plant-derived immunomodulatory component, this platform may also be adapted to other tissue models in which inflammation and regeneration are closely coupled. Such applications would require re-optimization of bioink rheology, cell compatibility, and lineage-specific culture conditions. The present protocol primarily models macrophage-mediated immune regulation and does not reproduce the full cellular complexity of the bone immune microenvironment. Neutrophils, osteoclasts, T cells, and other stromal populations were not systematically incorporated, and the use of nude mice limits the assessment of adaptive immune responses. Future models could incorporate endothelial cells and pericytes for prevascularization, monocytes or osteoclast precursors for bone-remodeling coupling, and temporally introduced neutrophils and T cells to reproduce sequential innate and adaptive immune responses. Multi-nozzle bioprinting or microfluidic culture could further support the spatial organization of bone-forming, vascular, and marrow-like compartments. Autologous human cell systems and immunocompetent animal models may provide additional opportunities to establish more physiologically representative multicellular bone organoids. Finally, the current evaluation period of up to 8 weeks in rodent critical-sized defects primarily reflects early-to-intermediate bone regeneration. Future studies should extend the observation period to 12–24 weeks and include serial micro-CT, quantification of residual scaffold, vascular and osteocyte maturation markers, and biomechanical testing. Validation in large-animal models will also be necessary to assess long-term scaffold turnover, vascular remodeling, bone maturation, and structural stability.

One possible application is the modeling of orthopedic diseases involving immune dysregulation. Patient-derived macrophages, synovial fibroblasts, osteoblast-lineage cells, or osteoclast precursors could be incorporated into modified constructs to study rheumatoid arthritis, osteoporosis, or other inflammatory bone disorders.^14^^,^^15^^,^^16^^,^^17^^,^^18^ Such models may help examine how immune-cell phenotypes influence the balance between bone formation and resorption under defined material and culture conditions.

This approach could also be extended to the engineering of other immune-responsive tissue organoids, such as cartilage and skin. For instance, incorporating well-established chondrogenic factors like TGF-β3 into the composite bioink would allow the generation of cartilage organoid systems. These models can support chondrocyte differentiation and enable the study of cartilage development in an immune-competent context. The process of the skin healing is based on the coordinated interaction of various immune cell types. The immunoregulatory features of this bioink may improve wound repair and reduce scar formation.^19^^,^^20^ Finally, given that the bone organoids fabricated with this technique simultaneously promoted bone formation and blood vessel growth *in vivo*, this bioink system could also be a tool to address the vascularization challenges commonly encountered in the *in vitro* engineering of other tissues and organs.

In conclusion, this approach represents a promising strategy for expanding the functional repertoire and enhancing the structural complexity of current *in vitro* organoid platforms. We anticipate that advances in these organoid technologies will be instrumental in deepening our knowledge of disease pathology, enabling the development of novel therapies and facilitating the clinical translation of functional implants (Figure 4).

**Figure 4:**
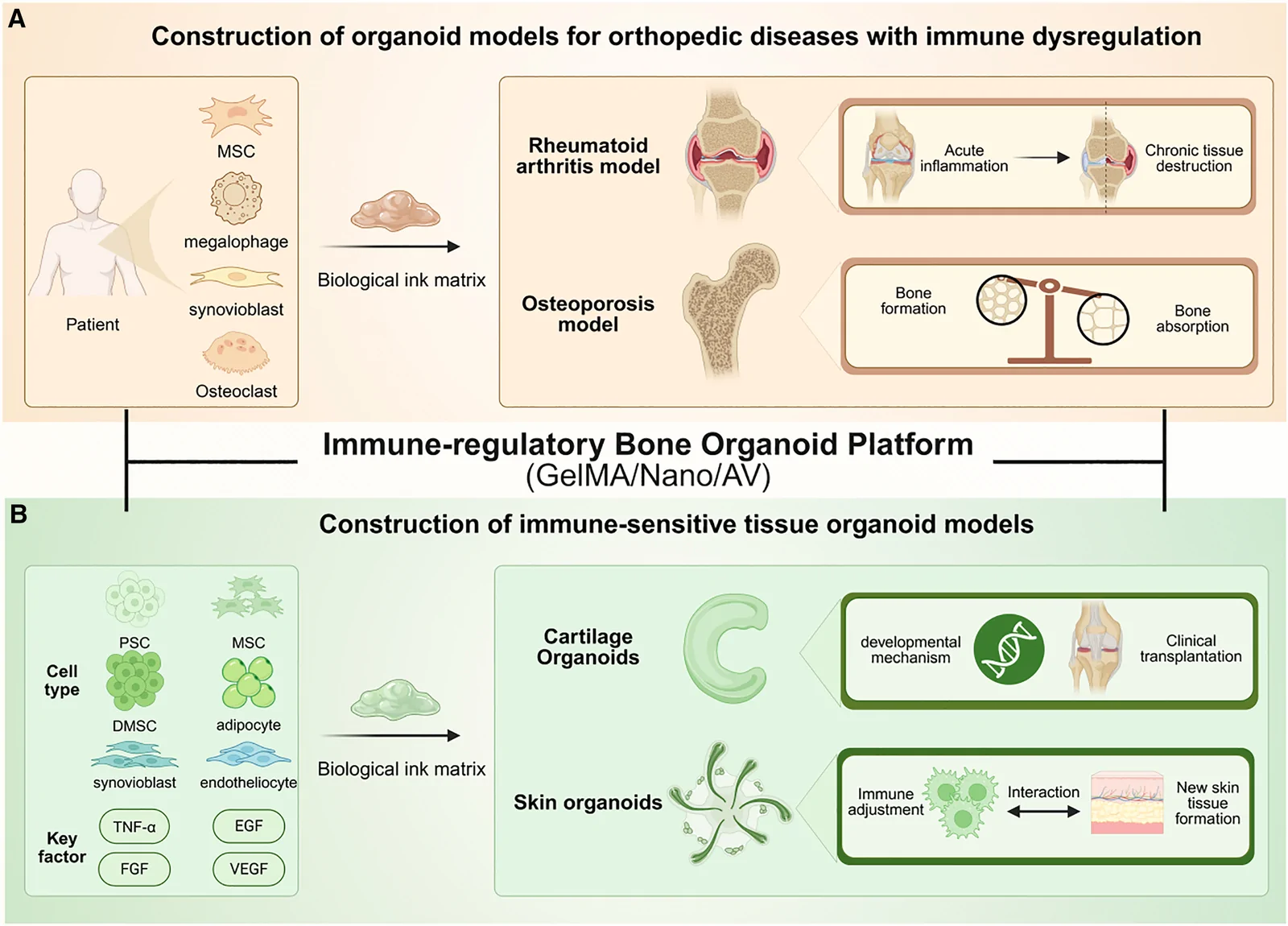
Potential future applications and extensions of the protocol (A) Construction of organoid models for orthopedic diseases with immune dysregulation. (B) Construction of immune-sensitive tissue organoid models.

### Limitations of the study

This protocol primarily models macrophage-mediated immune regulation and does not recapitulate the full cellular complexity of the bone immune microenvironment. Neutrophils, osteoclasts, T cells, and other stromal or immune populations were not systematically incorporated, and the use of nude mice limits evaluation of adaptive immune responses. The current maturation assessment focuses mainly on osteogenic differentiation, extracellular matrix deposition, and mineralization; late-stage osteocyte maturation and lacuno-canalicular organization were not systematically characterized. In addition, the proposed links between AV-associated bioactivity and immune signaling are based on phenotypic assays and bulk transcriptomic associations and therefore do not establish cell-specific or molecular causality. Finally, the *in vivo* evaluation period of up to 8 weeks primarily captures early-to-intermediate bone regeneration. Longer-term studies are required to assess scaffold degradation, vascular stabilization, biomechanical integration, and mature bone remodeling, and further validation in immunocompetent and large-animal models will be important for translation.

## Resource availability

### Lead contact

Requests for further information and resources should be directed to and will be fulfilled by the lead contact, Xiaoxiang Ren (rxx1991@shu.edu.cn).

### Materials availability

All unique/stable reagents generated in this study are available from the lead contact without restriction.

### Data and code availability

• All data reported in this paper will be shared by the lead contact upon request
• This paper does not report original code.
• Any additional information required to reanalyze the data reported in this paper is available from the lead contact upon request.

## Acknowledgments

The authors acknowledge the financial support from the 10.13039/501100001809National Natural Science Foundation of China (82427809, 32571554, 82202335, and 82230071), Fujian Province Natural Science Foundation project (2025J01300).

## Author contributions

T.W., G.G., X.Z., and K.H. contributed equally to this work. Conceptualization, T.W., X.R., and J.S.; methodology, G.G. and K.H.; investigation, T.W., X.Z., and C.L.; formal analysis, T.W. and X.R.; resources, C.H., G.W., J.S., and X.R.; writing, T.W. and X.R.; supervision, G.G., X.Z., K.H., and X.R.; funding acquisition, J.S. and X.R.

## Declaration of interests

The authors declare no competing interests.

## Declaration of generative AI and AI-assisted technologies in the manuscript preparation process

During the preparation of this work, the authors used Gemini for improving readability and language fluency. The authors reviewed and edited the output as needed and take full responsibility for the content of the published article.

## STAR★Methods

### Key resources table

**Table:** 

REAGENT or RESOURCE	SOURCE	IDENTIFIER
**Antibodies**		

Mouse monoclonal anti-Collagen Type I	Proteintech	Cat# 67288; RRID: AB_2882554
Rabbit polyclonal anti-RUNX2	Abcam	Cat# ab192256; RRID:AB_2713945
Mouse monoclonal anti-GAPDH	Proteintech	Cat# 60004; RRID: AB_2107436
Rabbit polyclonal anti-CD31	Abcam	Cat# ab28364; RRID: AB_726362
Rabbit polyclonal anti-Osteocalcin	Proteintech	Cat# 23418; RRID: AB_2879275
Rabbit monoclonal anti-Vinculin	Bioss	Cat# bsm-54148R
Rabbit monoclonal anti-Periostin	Bioss	Cat# bsm-54036R
Recombinant rabbit monoclonal anti-iNOS	Abcam	Cat# ab178945; RRID: AB_2861417
Rabbit polyclonal anti-CD206/MRC1	Affinity Biosciences	Cat# DF4149; RRID: AB_2836514
Alexa Fluor 488-conjugated anti-iNOS monoclonal antibody	Thermo Fisher Scientific	Cat# 53–5920-82; RRID: AB_2574443
PE-conjugated anti-mouse CD206 monoclonal antibody	BioLegend	Cat# 141701; RRID: AB_10900263
HRP-labeled secondary antibody	Yaenzyme	Cat# LF102

**Biological samples**		

Fresh leaves of Aloe vera var. barbadensis Miller	Multi Sciences (Lianke) Biotech	Cat# Customization

**Chemicals, peptides, and recombinant proteins**		

Gelatin methacryloyl (GelMA, substitution degree ∼60%)	This paper	N/A
Gelatin	Titan Scientific	Cat# Customization
Laponite-XLG nanoclay	BYK-Chemie GmbH	Cat# LAPONITE-XLG
Lithium phenyl-2,4,6-trimethylbenzoylphosphinate (LAP)	Engineering for Life (EFL)	Cat# EFL-LAP-01
Methacrylic anhydride (MA)	Aladdin	Cat# M102519
β-Glycerophosphate disodium salt pentahydrate	MedChemExpress (MCE)	Cat# HY-D0886
L-Ascorbic acid	MedChemExpress (MCE)	Cat# HY-B0166
Dexamethasone	MedChemExpress (MCE)	Cat# HY-14648
Trizol reagent	Invitrogen	Cat# 15596026
RIPA lysis buffer	Beyotime Biotechnology	Cat# P0013B
Protease inhibitor cocktail	Beyotime Biotechnology	Cat# P1045
Protein-free rapid blocking buffer	Yaenzyme	Cat# PS108
DAPI staining solution	Beyotime Biotechnology	Cat# C1006
Staining permeabilization solution (0.1% Triton X-100)	Beyotime Biotechnology	Cat# P0096
Crystal violet staining solution (0.5%)	Solarbio	Cat# G1062
EDTA decalcification solution (10 wt %, pH 7.2–7.4)	Servicebio	Cat# G1105
Anti-fluorescence quenching mounting medium	Servicebio	Cat# G1407

**Critical commercial assays**		

Calcein-AM/PI cell viability detection kit	Beyotime Biotechnology	Cat# C2015M
Cell Counting Kit-8 (CCK-8)	Beyotime Biotechnology	Cat# C0038
BCIP/NBT alkaline phosphatase chromogenic kit	Beyotime Biotechnology	Cat# C3206
Alizarin Red S staining solution	Beyotime Biotechnology	Cat# C0148
BCA protein assay kit	Beyotime Biotechnology	Cat# P0012
PrimeScript RT Master Mix	Takara Bio	Cat# RR036A
TB Green Premix Ex Taq (Tli RNaseH Plus)	Takara Bio	Cat# RR820A
Fast PAGE gel preparation kit	Yaenzyme	Cat# PG112
Omni-ECL chemiluminescence detection kit	Yaenzyme	Cat# SQ101
Hematoxylin and eosin (H&E) staining solution	Beyotime Biotechnology	Cat# C0105

**Experimental models: Cell lines**		

Rat: Bone marrow mesenchymal stem cells (rBMSCs), male SD rat origin	Cyagen Biosciences	Cat#RASMX-01001
Mouse: RAW 264.7: Monocyte/macrophage-like cell line, male BALB/c origin	Cyagen Biosciences	Cat# M3-0101

**Experimental models: Organisms/strains**		

Mouse: BALB/c nude: Athymic, female, 8 weeks old	Changzhou Cavens Laboratory Animal Co., Ltd.	N/A
Rat: Sprague-Dawley (SD): Wild-type, female, 8 weeks old	Changzhou Cavens Laboratory Animal Co., Ltd.	N/A

**Oligonucleotides**		

qPCR primers for target genes (IL-1β, CD206, iNOS, IL-10, RUNX2, OCN), see Table S1	This paper	N/A

**Software and algorithms**		

Autodesk 3ds Max 2020	Autodesk	https://www.autodesk.com/
GraphPad Prism 9	GraphPad Software	https://www.graphpad.com/
Origin 2021	OriginLab Corporation	https://www.originlab.com/
NRecon reconstruction software	Bruker Micro-CT	https://www.bruker.com/
CTAn morphometric software	Bruker Micro-CT	https://www.bruker.com/
CTVoX 3D visualization software	Bruker Micro-CT	https://www.bruker.com/
FlowJo v10.8	BD Biosciences	https://www.flowjo.com/
edgeR v3.38	Robinson et al.	https://bioconductor.org/packages/edgeR/
ImageJ (Fiji)	NIH	https://imagej.net/software/fiji/

**Other**		

3D bioprinter	Regenovo Biotechnology Co., Ltd.	cat. no. Bio-Architect SR
Micro-CT scanner	Skyscan	cat. no. SkyScan 1272
Confocal fluorescence microscope	Olympus	cat. no. FV3000
Real-time fluorescent PCR instrument	Analytik Jena	cat. no. qTOWER 3
Gel imaging system	Bio-Rad	cat. no. GelDoc XR+
Flow cytometer	Beckman	cat. no. CytoFLEX
Multifunctional microplate reader	BioTek	cat. no. Cytation 5
Freeze dryer	Labconco	cat. no. FreeZone 4.5

### Experimental model and study participant details

#### Primary cell cultures and cell lines

Rat bone marrow mesenchymal stem cells (rBMSCs, derived from male Sprague-Dawley rats) and the RAW 264.7 murine macrophage cell line (derived from a male BALB/c mouse) were purchased from Cyagen Biosciences (Guangzhou, China). rBMSCs were maintained in α-MEM supplemented with 10% fetal bovine serum (FBS) and 1% penicillin-streptomycin. Cells at passage 5 were harvested for bioink encapsulation and downstream osteogenic differentiation assays. RAW 264.7 macrophages were cultured in high-glucose DMEM supplemented with 10% FBS and 1% penicillin-streptomycin. All cultures were maintained at 37°C in a humidified atmosphere containing 5% CO_2_ and routinely tested to confirm the absence of mycoplasma contamination.

#### Nude mouse subcutaneous model

Eight-week-old female BALB/c nude mice (athymic) were purchased from Changzhou Cavens Laboratory Animal Co., Ltd. (Changzhou, China). Animals were housed in a specific-pathogen-free (SPF) facility under standardized environmental conditions (temperature: 22°C–24°C; relative humidity: 50%–60%; 12 h light/12 h dark cycle) with free access to a sterile standard rodent diet and water. Animals were randomly allocated to experimental groups for the ectopic subcutaneous implantation of bioprinted bone organoid precursors. All animal handling, surgical procedures, and post-operative care were approved by the Institutional Animal Care and Use Committee of Shanghai University (Approval No. ECSHU 2024-127) and performed in strict accordance with institutional animal care guidelines and ARRIVE standards.

#### Rat calvarial defect model

Eight-week-old female Sprague-Dawley (SD) rats (body weight: 200–220 g; total *n* = 48) were purchased from Changzhou Cavens Laboratory Animal Co., Ltd. (Changzhou, China). The rats were housed in the SPF barrier facility under controlled ambient conditions (temperature: 22°C–24°C; relative humidity: 50%–60%; 12 h light/12 h dark cycle) with *ad libitum* access to standard chow and water. Female animals were utilized across all cohorts to maintain physiological and baseline consistency in bone regeneration. Rats were randomly assigned to treatment and control cohorts (*n* = 6 per group at 4 and 8 weeks post-implantation). All surgical protocols, defect creation, and harvest procedures were approved by the Institutional Animal Care and Use Committee of Shanghai University (Approval No. ECSHU 2024-127) and complied with institutional and national regulations for laboratory animal use.

### Method details

#### Extraction of aloe vera gel

Fresh, mature leaves of Aloe vera var. barbadensis Miller were thoroughly washed with sterile deionized water and surface-sterilized under ultraviolet light for 1 h in a laminar flow hood. Under aseptic conditions, the external green epidermis was peeled using a sterile scalpel, and the inner parenchymatous gel was collected by scraping the hydrogel core. The crude gel was filtered through sterile gauze to remove particulate debris, stirred continuously at room temperature for 2 h to achieve uniform homogenization, and centrifuged at 8000 rpm for 15 min at room temperature to eliminate residual impurities. The clear supernatant was harvested as purified Aloe vera (AV) gel. Gel permeation chromatography (GPC) and liquid chromatography-mass spectrometry (LC-MS) were conducted to verify the polysaccharide profile and identify small-molecule bioactive constituents.

#### Synthesis and validation of GelMA

Gelatin methacryloyl (GelMA) was synthesized by dissolving 10 g of gelatin in 100 mL of deionized water under continuous stirring (300 rpm) at 50°C to prepare a 10 wt % solution. While maintaining 50°C, 4 mL of methacrylic anhydride was added dropwise at a feed rate of 0.5 mL/min, and the mixture was allowed to react under continuous stirring for 3 h. The reaction was terminated by adding 300 mL of deionized water. The solution was dialyzed against a 13 kDa cutoff membrane at 37°C for 7 days, replacing the deionized water 2–3 times daily. The completeness of dialysis was monitored electrochemically until the conductivity of the dialysate matched that of pure deionized water and the pH stabilized within the neutral range. The dialyzed solution was frozen in liquid nitrogen for 24 h and lyophilized for 72 h to yield a white porous GelMA powder. Complete removal of unreacted methacrylic anhydride and methacrylic acid byproducts and an approximately 60% degree of substitution were confirmed by ^1^H-NMR spectroscopy. Lyophilized GelMA was stored in a dry, light-shielded container at −20°C.

#### Preparation of plant-based composite bioinks

GelMA powder and Laponite nanoclay particles were sterilized under ultraviolet light for 2 h prior to bioink preparation. Three formulations were prepared under aseptic conditions.

1) GelMA bioink: 0.5 g of GelMA was dissolved in 5 mL of sterile deionized water containing 0.0125 g of lithium phenyl-2,4,6-trimethylbenzoylphosphinate (LAP) under continuous stirring at 37°C for 2 h to yield a 10 wt % GelMA bioink.
2) GelMA/Laponite bioink: 0.05 g of Laponite nanoclay was added to the 10 wt % GelMA solution and stirred at 37°C for 2 h until a homogeneous suspension was formed (10 wt % GelMA, 1 wt % Laponite).
3) GelMA/Laponite/AV bioink: 0.5 g of GelMA and 0.05 g of Laponite nanoclay were dispersed in 3 mL of sterile deionized water containing 0.0125 g of LAP. After 1 h of stirring at 37°C to ensure complete dispersion of Laponite without air bubbles, 2 mL of pre-prepared sterile Aloe vera gel was added and stirred for an additional 1 h at 37°C (final ratio: 10 wt % GelMA, 1 wt % Laponite, and 40 vol % AV).

#### 3D bioprinting of bone organoids precursors

Construct architecture was designed in Autodesk 3ds Max as an 8-layer rectangular porous grid (5 mm × 5 mm) with an infill spacing of 1.5 mm and an orthogonal 90° interlayer rotation applied twice. rBMSCs at passage 5 were detached with 0.25% trypsin-EDTA, centrifuged (1000 g, 5 min), and gently resuspended in pre-warmed (37°C) bioinks at a density of 1.0×10^6^ cells/mL. The cell-laden bioink was loaded into a light-shielded cartridge and printed using a low-temperature extrusion bioprinter (Bio-Architect SR) at 8°C. Printing parameters were set to: pneumatic pressure of 0.2–0.3 MPa, printing speed of 4 mm/s, retraction height of 2 mm, and a 21G metal nozzle. Filaments were crosslinked layer-by-layer under 365 nm UV light at 5% intensity (20 mW/cm^2^) for 10 s per layer, followed by 30 s post-curing to stabilize the construct. Printability (Pr) and diffusion rate (Dfr) were calculated from stereomicrographs:

$$\mathrm{Pr}=\frac{\pi }{4}\times \frac{1}{c}=\frac{L^{2}}{16A}$$

$$Dfr=\frac{{At}^{f}-{Aa}^{f}}{{Aa}^{f}}\times 100\%$$

where L is the pore perimeter, A is the pore area, C is circularity, At^f^ is the theoretical pore area, and Aa^f^ is the actual pore area.

#### *In vitro* culture of bone organoids

Immediately following printing, construct precursors were transferred into multi-well plates and incubated at 37°C in a humidified 5% CO_2_ atmosphere. Constructs were cultured in growth medium (α-MEM containing 10% FBS and 1% penicillin-streptomycin) for the first 24 h. The medium was subsequently replaced with osteogenic induction medium consisting of α-MEM supplemented with 10% FBS, 1% penicillin-streptomycin, 10 mM β-glycerophosphate, 50 μg/mL L-ascorbic acid, and 100 nM dexamethasone. Cultures were maintained under static conditions for up to 28 days, with medium refreshed every 2–3 days.

#### Cell viability and proliferation assays

Cell viability within bioprinted constructs was assessed at days 1, 4, 7, and 14 using a Calcein-AM/propidium iodide (PI) detection kit. Constructs were washed three times with PBS and incubated with staining solution (Calcein-AM: PI: PBS = 1 μL: 1 μL: 1000 μL) at 37°C for 30 min in the dark. After three PBS washes, fluorescence images were acquired using a confocal laser scanning microscope (Olympus FV3000), and live/dead ratios were quantified using ImageJ software. Proliferation of encapsulated rBMSCs was evaluated on days 1, 4, 7, and 14 using a CCK-8 kit. Constructs were rinsed with PBS and incubated in 10% CCK-8 working solution prepared in culture medium at 37°C for 1 h protected from light. A 100 μL aliquot of the incubated supernatant was transferred into a 96-well plate, and absorbance at 450 nm was measured on a microplate reader.

#### Alkaline phosphatase and Alizarin Red S staining

For 2D co-culture evaluation, rBMSCs were seeded in 12-well plates and co-cultured with acellular scaffolds placed in Transwell inserts, using growth medium on day 1 and osteogenic induction medium from day 2 onward. On day 10, cells were fixed with 4% paraformaldehyde (PFA) for 15 min and washed with PBS. Alkaline phosphatase (ALP) staining was performed using a BCIP/NBT chromogenic kit for 30 min according to the manufacturer’s protocol. For Alizarin Red S (ARS) staining, fixed cells were stained with ARS solution for 30 min and rinsed with PBS. For 3D bone organoids, constructs cultured for 14 and 28 days were fixed in 4% PFA for 15 min, embedded in paraffin wax, and sliced into 5 μm thick sections. Deparaffinized sections were stained with ARS or BCIP/NBT ALP staining solutions for 30 min, washed three times with PBS, and imaged on a microplate reader and optical microscope.

#### Gene expression analysis by RT-qPCR

On day 10 of osteogenic co-culture, cells were lysed by adding 500 μL of Trizol reagent per well, rocked to ensure complete coverage, and incubated on ice for 5 min. Chloroform (100 μL) was added, vortexed vigorously until milky-white, held on ice for 5 min, and centrifuged at 12000 g for 15 min at 4°C. The clear upper aqueous layer was transferred into a fresh microcentrifuge tube, mixed with an equal volume of isopropanol, incubated on ice for 10 min, and centrifuged at 12000 g for 10 min at 4°C. The RNA pellet was washed with 1 mL of 75% ethanol, centrifuged at 7500 g for 5 min at 4°C, air-dried for 10 min, and dissolved in RNase-free DEPC water. Concentration and purity were measured using a microplate reader. Complementary DNA was synthesized from 1000 ng of total RNA in a 20 μL reaction containing PrimeScript RT master mix (37°C for 15 min, 85°C for 15 s, and held at 4°C). RT-qPCR was performed using TB Green Premix Ex Taq on a qTOWER 3 system in a 20 μL reaction containing diluted cDNA and specific primers. Relative target gene expression was determined using the 2^−ΔΔCt^ method normalized against GAPDH.

#### Protein analysis by western blot

Following 10 days of osteogenic co-culture, adherent cells were washed with PBS and lysed in 200 μL of RIPA buffer containing protease inhibitors, phosphatase inhibitors, and EDTA on ice for 30 min. Lysates were centrifuged at 12000 g for 5 min at 4°C, and total protein concentration was measured using a BCA kit. Protein samples were mixed with 5× protein loading buffer at a 4:1 volume ratio and denatured at 100°C for 10 min. Equal amounts of protein (25 μg/lane) were loaded onto 10% SDS-PAGE gels, resolved at 80 V for 30 min and 120 V for 60 min, and electrotransferred onto methanol-activated PVDF membranes at 300 mA for 100 min in an ice-cooled tank. Membranes were blocked with protein-free rapid blocking buffer for 15 min and incubated overnight at 4°C with primary antibodies against Collagen type I (1:1000), RUNX2 (1:1000), and GAPDH (1:2000). Membranes were washed three times with TBST (10 min each) and incubated with HRP-conjugated secondary antibodies (1:5000) for 1.5 h at room temperature. Bands were visualized using an Omni-ECL chemiluminescence kit on a Bio-Rad imaging system.

#### Micro-CT scanning and analysis

Mineralization of *in vitro* constructs and *in vivo* bone repair was characterized using a SkyScan micro-CT scanner (SkyScan 1172/1272). Scanning parameters for subcutaneously implanted and in vitro-cultured bone constructs were adjusted to 65 kV and 80 μA, with an isotropic voxel size of 9 μm, a 0.5 mm aluminum filter to minimize beam hardening, and a rotation step of 0.4° over a total rotation of 360°. Cross-sectional images were reconstructed using NRecon software and analyzed with CTAn software. A uniform circular region of interest (ROI) centered on the construct was defined to encompass the volume. Bone volume fraction (BV/TV %), trabecular thickness (Tb.Th, μm), and trabecular number (Tb.N, 1/mm) were determined using auxiliary system software. Three-dimensional images were reconstructed using CTVoX software.

#### Macrophage recruitment and invasion

A Transwell co-culture system was used to evaluate macrophage migration. RAW 264.7 cells (1 × 10^5^ cells) were seeded into the upper insert, while bone organoid scaffolds were placed in the lower chamber. The co-culture was incubated at 37°C for 24 h. Non-migrated cells remaining on the upper surface were removed using a sterile cotton swab, and cells that traversed the membrane to the underside were fixed with 4% PFA and stained with 0.5% crystal violet solution. For invasion assays, the upper insert was pre-coated with 0.1 wt % gelatin and incubated overnight at 4°C prior to seeding RAW 264.7 cells. Migrated and invaded cells were observed and imaged under an optical microscope.

#### Macrophage adhesion and polarization

For cell adhesion, 1 × 10^4^ RAW 264.7 cells were seeded onto the surface of printed acellular scaffolds in multi-well plates. On days 1, 4, and 7, scaffolds were retrieved and subjected to Calcein-AM/PI viability staining. For macrophage polarization, RAW 264.7 cells (1 × 10^4^ cells) were seeded on coverslips in the lower Transwell chamber, while scaffolds were placed in the upper insert. After 24 h of co-culture, coverslips were fixed with 4% PFA for 15 min, permeabilized with immunofluorescence permeabilization reagent (0.1% Triton X-100) for 15 min, blocked with ready-to-use goat serum for 30 min, and incubated with primary antibodies against iNOS (1:500) and CD206 (1:500) overnight at 4°C. Coverslips were then incubated with Alexa Fluor 488- and Alexa Fluor 594-conjugated secondary antibodies for 30 min, counterstained with DAPI for 5–20 min in the dark, and imaged on a confocal laser scanning microscope (Olympus FV3000). The sustained regulatory effect on macrophages was similarly evaluated by co-culturing scaffolds retrieved after 14 and 28 days of culture.

#### Flow cytometry analysis of macrophages

Following 24 h of Transwell co-culture, RAW 264.7 cells were detached with trypsin-EDTA, washed with PBS, and collected by centrifugation (1000 g, 5 min). Cells were fixed in 4% PFA for 10 min, permeabilized with 0.1% Triton X-100 for 10 min, and incubated with Alexa Fluor 488-conjugated anti-iNOS (1:100) and PE-conjugated anti-CD206 (1:100) antibodies for 30 min. After repeated washing with PBS, cells were analyzed on a flow cytometer to determine the percentages of iNOS^+^ and CD206^+^ populations.

#### Subcutaneous implantation in nude mice

Eight-week-old female BALB/c nude mice were anesthetized with isoflurane (induction: 3%–5%, maintenance: 1%–2%). The dorsal region was disinfected with povidone-iodine, a midline incision was made, and bone organoid precursors were implanted subcutaneously before closing the incision. At 2 and 4 weeks post-surgery, mice were euthanized, and implants were retrieved for Micro-CT analysis, H&E staining, and immunofluorescence. Constructs were fixed in 4% PFA and decalcified in 10 wt % EDTA solution (pH 7.2–7.4) for 4 weeks. Sections (5 μm) were prepared from paraffin-embedded tissues and processed for H&E staining or blocked with 3% BSA for immunofluorescence staining. Sections were incubated overnight at 4°C with primary antibodies (OCN, RUNX2, Periostin, CD31, Collagen I, Vinculin, iNOS, and CD206, each diluted 1:500 in 3% BSA), followed by secondary antibody incubation (1:500 in 3% BSA) and confocal imaging.

#### Bulk RNA sequencing and analysis

Dorsal subcutaneous bone organoids harvested at 4 weeks post-surgery were subjected to RNA sequencing. Total RNA was isolated using Trizol, and polyadenylated mRNA was enriched using magnetic oligo(dT) beads and thermally fragmented. Fragments were reverse-transcribed into first-strand cDNA, followed by second-strand cDNA synthesis, end repair, 3′ end adenylation, adapter ligation, and RT-qPCR amplification. Sequencing libraries were sequenced on an Illumina platform. Biological replicate reproducibility was assessed using Pearson correlation coefficients in R. Differential expression analysis was performed using edgeR. Gene Ontology (GO) and Kyoto Encyclopedia of Genes and Genomes (KEGG) analyses were performed using hypergeometric tests to evaluate pathway enrichment among differentially expressed genes.

#### Rat calvarial defect repair surgery

Eight-week-old female SD rats were anesthetized by intraperitoneal injection of 1% chloral hydrate. The cranium was shaved and disinfected with povidone-iodine. A midline scalp incision was made, and bilateral 5 mm critical-sized calvarial defects were created using a circular trephine drill under continuous saline irrigation, taking care to avoid injury to the dura mater.^21^ Bone organoid precursors were implanted into the defect sites, and the incision was sutured. Rats were euthanized at 4 and 8 weeks post-implantation via an overdose of pentobarbital sodium. Cranial specimens were harvested for Micro-CT scanning, decalcified in 10 wt % EDTA solution, sectioned, and processed for H&E staining and immunofluorescence staining (OCN, Runx2, CD206, and iNOS).

#### *In vivo* biosafety evaluation

At 8 weeks post-implantation, major internal organs (heart, liver, spleen, lungs, and kidneys) were harvested from SD rats, fixed in 4% PFA for 24–48 h, dehydrated, paraffin-embedded, sectioned at 5 μm thickness without decalcification, and stained with H&E to evaluate histological biosafety. Blood samples were collected at 4 weeks post-operation for routine hematological counts (white blood cells, red blood cells, and monocytes) and serum biochemical analysis (ALT, AST, BUN, and CREA).

### Quantification and statistical analysis

Image processing and morphometric analysis.
