# Supplementary material for: Protocol for fabricating immune-regulatory bone organoids via plant-based bioinks
Source: iScience. 2026 Oct 12;29(11):117866. doi: 10.1016/j.isci.2026.117866 (PMC13682476; doi:10.1016/j.isci.2026.117866)
Supplement: Supplementary file 1 [file mmc1.pdf]

iScience, Volume *29*

Supplemental information

Protocol for fabricating immune-regulatory bone organoids via plant-based bioinks

Tianhao Wang, Gan Gao, Xiaoxu Zhang, Kaifeng Huo, Chenglong Liu, Zhen Geng, Long Bai, Hao Zhang, Han Liu, Ke Xu, Yuxiao Lai, Jianhua Wang, Yingying Jing, Chongru He, Guangchao Wang, Jiacan Su, and Xiaoxiang Ren

Supplemental Information

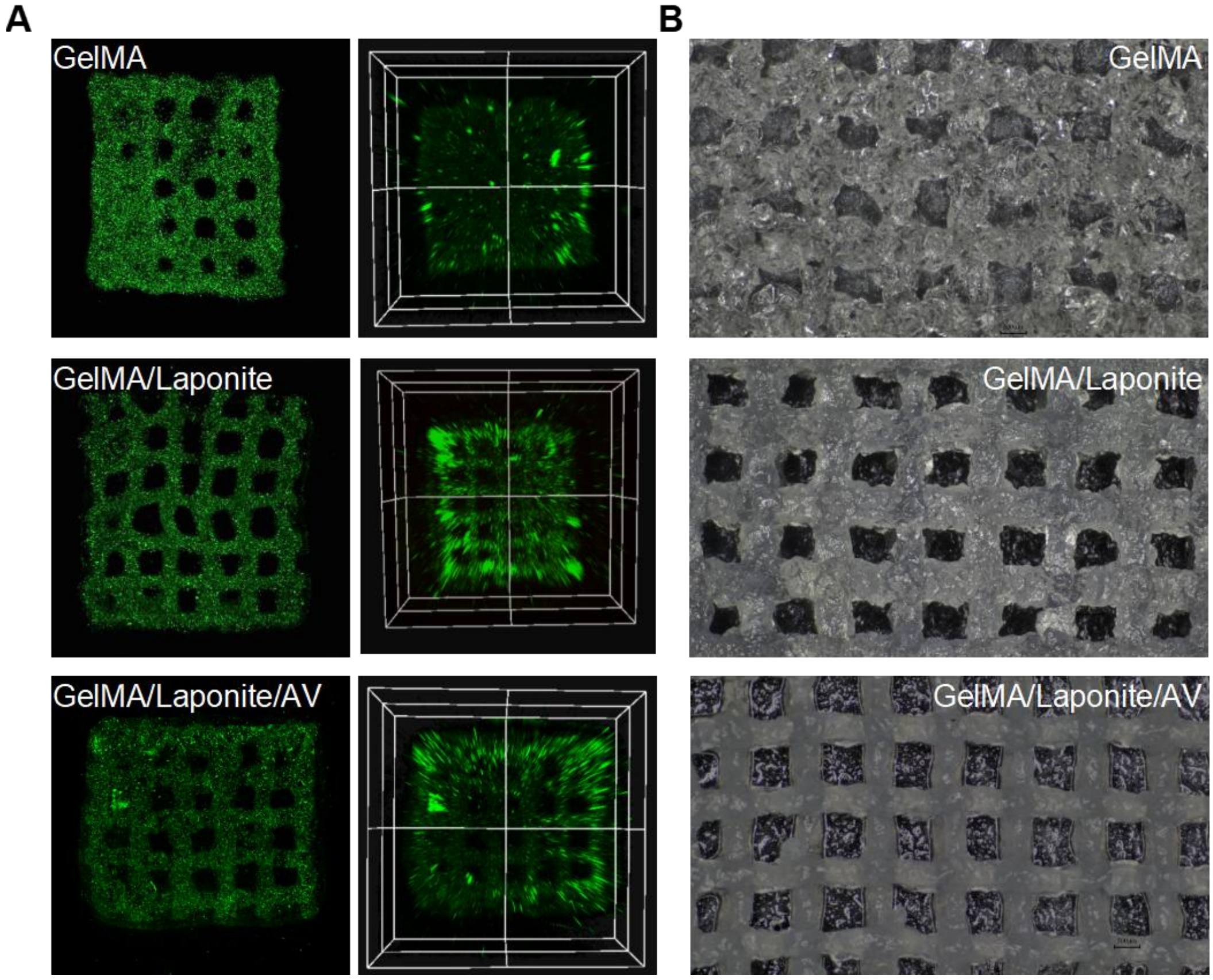

Figure S1. In vitro characterization and structural fidelity of 3D bioprinted bone organoid precursors. A) Fluorescence images of live/dead staining showing cell viability within 3D bioprinted bone organoid precursors. B) Bright-field images displaying the macro-morphology and structural fidelity of 3D bioprinted constructs. Reproduced with permission.[S1] Copyright 2026, Elsevier Ltd.

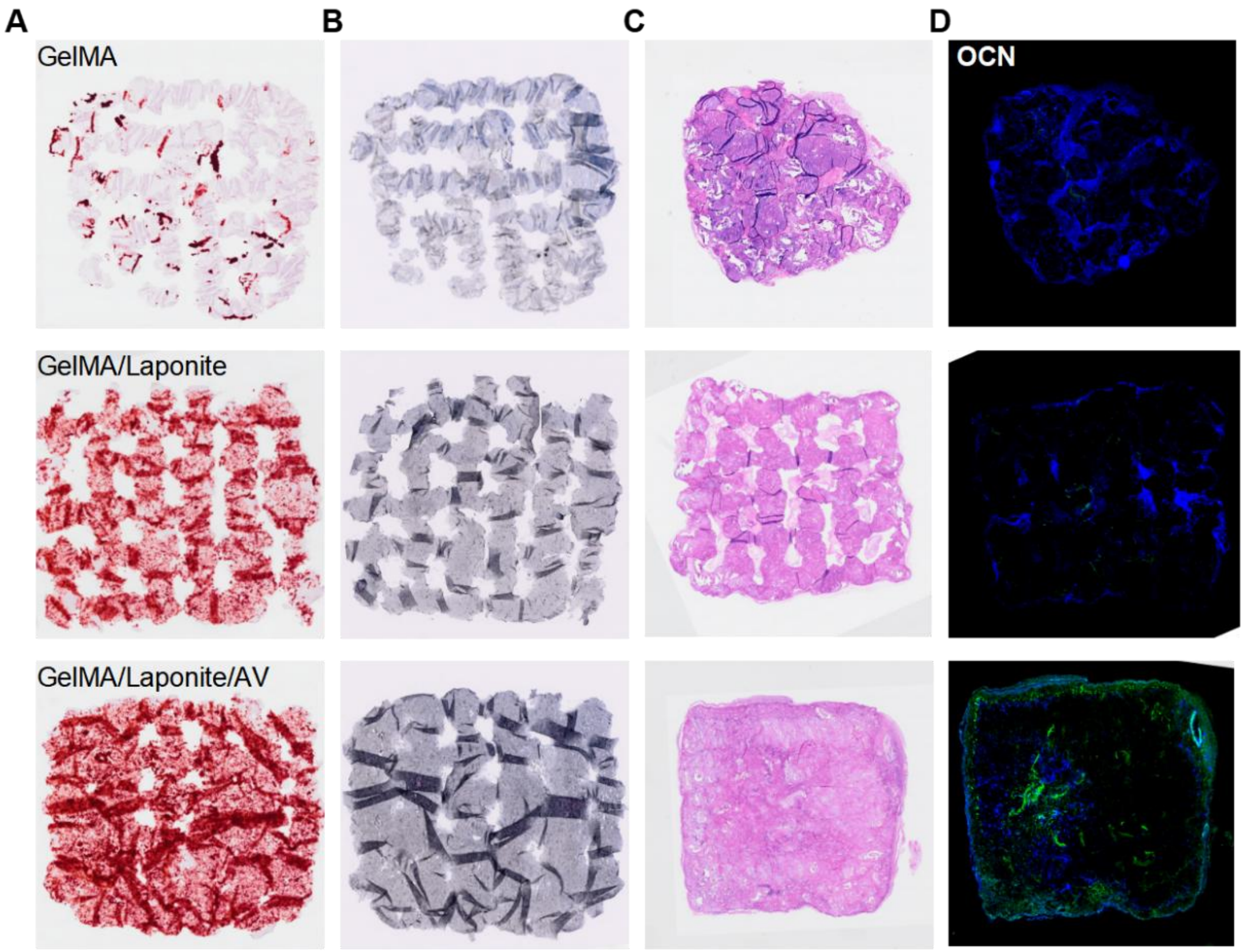

Figure S2. Osteogenic differentiation and in vivo histological evaluation of bone organoids. A) Representative Alizarin Red S (ARS) staining. B) Alkaline Phosphatase (ALP) staining of bone organoids cultured in vitro for 28 days. Reproduced with permission. C) H&E staining in bone organoids after 28 days of in vivo development. D) immunofluorescence staining for OCN in bone organoids after 28 days of in vivo development. Reproduced with permission.[S1] Copyright 2026, Elsevier Ltd.

Table S1. Primer sequences used for quantitative real-time PCR analysis

Primer Designation	Primer Sequence
IL-1β-F	TGGAGAGTGTGGATCCCAAG
IL-1β-R	GGTGCTGATGTACCAGTTGG
CD206-F	CTCTGTTCAGCTATTGGACGC
CD206-R	CGGAATTTCTGGGATTCAGCTTC
iNOS-F	GCGCTCTAGTGAAGCAAAGC
iNOS-R	ACTGCCTTCACTCTGCATTTG
IL-10-F	GAGAAGCATGGCCCAGAAATC
IL-10-R	GAGAAATCGATGACAGCGCC
RUNX2-F	GACTGTGGTTACCGTCATGGC
RUNX2-R	ACTTGGTTTTTCATAACAGCGGA
OCN-F	AGTGTGAGCTTAACCCTGCT
OCN-R	GAGGATCAAGTCCCGGAGAG

Supplemental References

S1. Zhang, Y., Wang, F., Li, R., Huang, D., Gao, Q., Zhang, Q., Geng, Z., Bai, L., Wang, J., Jing, Y., et al. (2026). Immune-instructive bone organoids via non-immunogenic plant-based bioinks for enhanced bone repair. Matter 9(4), 102651. https://doi.org/10.1016/j.matt.2026.102651.
